# Prognostic value of lactate in cats presented in respiratory distress to the emergency room

**DOI:** 10.3389/fvets.2022.918029

**Published:** 2022-09-13

**Authors:** Cassandra Gilday, Liz Guieu

**Affiliations:** ^1^Department of Small Animal Clinical Sciences, University of Tennessee College of Veterinary Medicine, Knoxville, TN, United States; ^2^Department of Small Animal Clinical Sciences, North Carolina State University College of Veterinary Medicine, Raleigh, NC, United States; ^3^Department of Small Animal Clinical Sciences, Colorado State University College of Veterinary Medicine, Fort Collins, CO, United States

**Keywords:** feline, biomarker, hyperlactatemia, lactate clearance, outcome, emergency, heart failure

## Abstract

Studies demonstrating the prognostic utility of plasma lactate concentration and lactate clearance in cats are limited. The objective of this study was to determine the prognostic utility of plasma lactate concentration upon admission and plasma lactate clearance in cats presented to the emergency room with respiratory distress attributed to underlying cardiac or respiratory disease. Additionally, we sought to determine if plasma lactate concentration on admission was significantly associated with the underlying cause of respiratory distress (cardiac vs. respiratory), type of respiratory disease, and vital parameters. Seventy-one cats presented in respiratory distress to the ER at a university teaching hospital were enrolled in this retrospective study. Admission lactate concentration was not associated with survival, duration of hospitalization, vital parameters, or underlying etiology for respiratory distress. In contrast, lactate clearance was significantly associated with survival and length of hospitalization. While a statistically significant association between lactate clearance and length of hospitalization was identified, this finding lacked clinical significance where an increase in lactate concentration by 1% was associated with an increased length of hospitalization by 11.4 min (*p* = 0.035). Results of this study suggest that lactate clearance may have prognostic utility in this population of cats. Further studies including the larger population of cats with underlying cardiac or respiratory disease are warranted.

## Introduction

Plasma lactate measurements are widely used as a metabolic marker of tissue hypoxia (1). Lactate studies in small animal veterinary medicine have been primarily performed on dogs. Multiple studies have found admission plasma lactate concentration to have prognostic utility in dogs with trauma, sepsis and/or systemic inflammatory response syndrome, spontaneous hemoabdomen, gastric dilation and volvulus, immune-mediated hemolytic anemia, babesiosis, and heartworm caval syndrome (2–11). Serial lactate concentrations may be more predicative of survival than a single measurement in dogs and may provide a more effective way to monitor response to treatment (8, 9, 12–14). In particular, persistently high lactate concentrations are significantly associated with increased mortality (13).

Studies evaluating the prognostic utility of lactate in cats are limited. This may be due in part to concerns that stress can induce hyperlactatemia in healthy cats affecting its reliability as a biomarker (15). However, this point remains controversial as a recent study found that the lactate concentration was not affected by struggling during venipuncture, venipuncture site, age, sex, or time after admission in healthy cats, suggesting it may have value as a prognostic indicator in this population (16). Furthermore, hyperlactatemia has been shown to be significantly associated with abnormal perfusion parameters in cats evaluated by an emergency service (17).

Studies investigating the prognostic utility of lactate in cats have had conflicting results. The prevalence of hyperlactatemia has ranged from 23 to 67% in cats presenting to the emergency room (14, 18, 19). Admission lactate concentration was not associated with survival in 111 cats presenting to an emergency service (17). Similarly, admission and serial lactates were not associated with survival or length of hospitalization in a population of 123 cats hospitalized for emergency care at a private referral center (18). In contrast, Kohen *et al*. reported hyperlactatemic cats had a significantly higher mortality rate (44.6%) when compared with cats that were normolactatemic (22.6%) in a population of 185 cats presenting to the emergency room (19). Similarly, in 444 cats presenting to the emergency room, hyperlactatemia on intake and lactate clearance were significantly associated with the outcome (14).

Hyperlactatemia has been associated with specific disease states and outcomes in multiple species (2). To these authors' knowledge, admission and serial lactate measurements have not been evaluated as a predictor of survival or length of hospitalization in cats presented with respiratory distress secondary to underlying cardiac or respiratory disease.

The primary goal of this study was to determine the prognostic utility of plasma lactate concentration upon admission, as well as serial lactate measurements in cats presented with respiratory distress to the emergency room. Our secondary aim was to determine if plasma lactate concentration at admission could help differentiate cats with an underlying cardiac vs. respiratory disease. Furthermore, among cats with an underlying respiratory disease, we seek to identify any difference in lactate concentration at admission based on the sub-categorization of respiratory disease. Finally, we aimed at identifying any association between vital parameters (respiratory rate, heart rate, systolic blood pressure, temperature, SpO_2_) and plasma lactate concentration upon admission. We hypothesized that hyperlactatemia at admission (blood lactate concentration ≥ 2.5 mmol/L) or an increase in lactate concentration during hospitalization would reflect more severe tissue hypoxia and failure to respond to therapy leading to longer hospitalization and a higher mortality rate.

## Materials and methods

### Case selection criteria

The medical record database of the University of Tennessee Veterinary Teaching Hospital was searched to identify cats that were admitted on an emergency basis to the ICU and placed in the oxygen cage over a 22-month period (January 2019 to October 2020). To be eligible for inclusion in this study, cats that were presented in respiratory distress were required to have had an admission, blood lactate concentration measured, as well as diagnostic imaging [i.e., thoracic radiographs, thoracic CT, echocardiogram, Thoracic Point of Care Ultrasound (T-POCUS)] to confirm the underlying cause of their respiratory distress. Cats were excluded when the underlying cause of respiratory distress could not be clearly categorized as respiratory or cardiac in origin.

### Medical records review

Data collected from the medical record included signalment (age, sex, neuter status, breed), blood lactate concentration, body weight, vitals (respiratory rate, heart rate, temperature, mucous membranes, capillary refill time), and physical exam findings (respiratory pattern, pulse quality) at admission.

The following bed-side diagnostic test findings were also recorded when available: systolic blood pressure (Doppler), Thoracic Point of Care Ultrasound (T-POCUS) findings, pulse oximetry (SPO_2_), PCV/TS, and blood glucose concentration. When available, recheck lactate concentration obtained at 7 am the following morning was recorded, as well as the time (in hours) lapsed from the initial measurement. In these cases, percent clearance was calculated as follows: ([(initial lactate- recheck lactate)/initial lactate] ×100) (11). For statistical purposes, tachypnea was defined as a respiratory rate > 40 breaths per minute (brpm), hypothermia was defined as a temperature <100°F (37.8°C), fever was defined as a temperature > 103°F (39.4°C), bradycardia was defined as a heart rate <160 beats per minute (bpm), tachycardia was defined as a heart rate > 225 bpm, hypotension was defined as a systolic blood pressure ≤ 90 mmHg, and hypoxemia was defined as SPO_2_ <95% (17). Hyperlactatemia was defined as a blood lactate concentration > 2.5 mmol/L and normolactatemia as a blood lactate concentration ≤ 2.5 mmol/L (14, 16–19). The results of all other pertinent diagnostic tests, including thoracic radiographs, T-POCUS, echocardiogram, thoracic CT, fluid analyses, fine needle aspirates, and necropsy results, were recorded when available. Clinical diagnosis, duration of hospitalization, and outcome (survival vs. non-survival) were also extracted from the medical record. Survivors were defined as cats that were discharged from the hospital. Non-survivors were defined as cats that either died or were euthanized in the hospital.

Cats were then categorized into two groups based on the underlying cause of their respiratory distress: cardiac vs. respiratory disease. The majority of cats categorized in the “cardiac disease” group had an echocardiogram and/or thoracic radiographs confirming congestive heart failure and an underlying cardiac disease at the time of inclusion. A minority of cats, previously diagnosed with underlying cardiac disease (within a year), remained eligible for inclusion if they were presented in respiratory distress, had an enlarged La:Ao (>1.5) and B-lines and/or pleural effusion identified on T-POCUS and responded to the furosemide therapy, even though advanced imaging was not repeated at the time of inclusion. All cats deemed to have a respiratory disease as the underlying cause for their respiratory distress were required to have diagnostic imaging (i.e., thoracic radiographs and/or thoracic CT) performed at that visit. Additional diagnostics to further characterize the underlying respiratory disease (i.e., pleural fluid analysis, endo/trans-tracheal wash, etc.) were not required for inclusion. Based on results of thoracic radiographs and/or thoracic CT, the respiratory disease was further subcategorized into upper airway (i.e., pharynx, larynx, nasal cavity, tracheal), lower airway (i.e., bronchi), lung parenchyma, pleural space, or > 1 disease localization.

### Statistical analysis

Continuous variables were assessed for normality by the Shapiro-Wilk test. Normally distributed parameters were reported using mean and standard deviation. Median and range were used for non-normally distributed parameters. The Mann–Whitney *U*-test was used to compare between two groups, and the Kruskal-Wallis test was used to compare among more than two groups. The Spearman rank correlation was used to assess the correlation between two continuous variables. Association between two groups of categorical data was evaluated using the Chi-square test or Fisher's Exact test. The logrank survival analysis was performed to further explore whether blood lactate concentration affects the survival distribution of the duration of hospital stay. The robust regression was used to further explore the significant correlation between the percentage change of lactate (predictor) and the length of stay days (dependent variable). Values of *p* ≤ 0.05 were considered significant for all comparisons. SAS software, version 9.4, release TS1M6 was used for all the analyses.

## Results

One hundred and twenty-one cats were initially identified as being admitted on an emergency basis to the ICU and placed in the oxygen cage. Fifty cases were excluded for the following reasons: absence of respiratory distress on presentation (*n* = 10), admission blood lactate concentration not available (*n* = 15), underlying cause of the respiratory distress was not cardiac or respiratory in origin (i.e., anemia) (*n* = 5), and unclear cause of respiratory distress (*n* = 20).

Seventy-one cases were enrolled in this study. The mean age of our population was 8.9 ± 4.8 years and the mean body weight was 5.1 ± 2.1 kg. Twenty-four cats were spayed females (34%), two were intact females (3%), 44 were neutered males (62%), and one was an intact male (1%). Breeds included Domestic Short Hair (*n* = 46, 65%), Domestic Long Hair (*n* = 8, 11%), Domestic Medium Hair (*n* = 4, 6%), mixed breed cat (*n* = 5, 7%), Siamese (*n* = 3, 4%), Maine Coon (*n* = 2, 3%), and one each of Bengal, Exotic Shorthair, and Himalayan. Physical examination findings are summarized in Table 1. Admission lactate concentration was 2.5 mmol/L (0.6–12.8). Thirty-eight cats (54%) had an admission lactate concentration ≤ 2.5 mmol/L, while 33 (46%) had an admission lactate concentration > 2.5 mmol/L. Recheck lactate concentration taken within 13.2 ± 5.2 h of admission was 1.7 ± 0.9 mmol/L with an associated percent clearance of 26.6 ± 45.4%. Thirty-six cats (51%) had underlying cardiac disease while 35 (49%) had underlying respiratory disease. The 35 cats with underlying respiratory disease were further subcategorized into upper airway (*n* = 4, 11%), lower airway (*n* = 9, 26%), parenchymal (*n* = 7, 20%), pleural space disease (*n* = 7, 20%), or disease localized to more than one category (*n* = 8, 23%). The length of hospitalization was 1 (0.5–12) day. Fifty-four cats (76%) survived to discharge and 17 (24%) did not [16 (94%) were euthanized, 1 (6%) died in hospital].

**Table 1:** Vital parameters and admission lactate concentration (mmol/L) in a population of 71 cats presented in respiratory distress to the emergency room.

	***N***	**Results**	Lactate^*^	***p*-value**	**Lactate ≤ 2.5^°^*N*, (%)**	**Lactate > 2.5^°^*N*, %**	***p*-value**
RR	67	70.8 ± 26.8	0.6–12.8 (2.5)	0.77			–
	56	>40	0.6–12.8 (2.5)	–	30 (44.8)	26 (38.8)	1.00
	11	≤ 40	1.1–6.1 (2.1)		6 (9)	5 (7.5)	
T (°F)	62	100.2 ± 2.4	0.6–12.8 (2.5)	0.88			–
	35	100–103	0.6–12.8 (2.4)	–	19 (30.7)	16 (25.8)	0.85
	22	<100	0.9–6.1 (2.5)		14 (22.6)	8 (12.9)	
	5	>103	1.9–3.1 (2.5)		3 (4.8)	2 (3.2)	
HR (bpm)	70	192.4 ± 39.7	0.6–12.8 (2.5)	0.62			–
	43	160–225	0.6–12.8 (2.5)	–	24 (34.3)	19 (27.1)	0.42
	14	<160	1–6.1 (2.4)		9 (12.9)	5 (7.1)	
	13	>225	0.9–5.7 (2.7)		5 (7.1)	8 (11.4)	
SBP^∧^ (mmHg)	34	124.1 ± 37.3	0.6–12.8 (2.5)	0.07			–
	5	≤ 90	2.5–12.8 (2.7)	–	2 (5.9)	3 (8.8)	0.35
	29	>90	0.6–6.1 (2.1)		19 (55.9)	10 (29.4)	
SPO_2_ (%)	14	98 (70–100)	1.1–3.6 (2.0)	0.85			–
	8	≥95	1.1–3.6 (2.2)	–	4 (25.6)	4 (28.6)	0.30
	6	<95	1.7–3.6 (2)		5 (35.7)	1 (7.1)	

* When lactate is considered as a continuous variable.

° When lactate is considered as a categorical variable with lactate ≤ 2.5 mmol/L being normal and lactate > 2.5 mmol/L being hyperlactatemia.

∧ Systolic blood pressure measured *via* Doppler.

RR, respiratory rate; T, temperature; HR, heart rate; bpm, beats per minute; SBP, systolic blood pressure; mmHg, millimeters of mercury; SPO_2_, pulse oximetry.

### Association between vital parameters, blood pressure, pulse oximetry, and admission blood lactate concentration

Physical examination findings are summarized in Table 1. When considered as continuous variables, respiratory rate (*p* = 0.77), temperature (*p* = 0.88), heart rate (*p* = 0.62), systolic blood pressure (*p* = 0.07), and SPO_2_ (*p* = 0.85) were not significantly associated with admission lactate concentration (Table 1). When considered as categorical variables, respiratory rate (>40, ≤ 40 brpm) (*p* = 1.0), temperature (>103, 100–103, <100°F) (*p* = 0.85), heart rate (<160, 160–225, >225 bpm) (*p* = 0.42), systolic blood pressure ( ≤ 90, >90 mmHg) (*p* = 0.35), and pulse oximetry (≥95, <95%) (*p* = 0.30) were not significantly associated with lactate concentration on admission (Table 1).

### Prognostic utility of plasma lactate concentration upon admission

Lactate on presentation for our population was 2.5 (0.6–12.8) mmol/L with hyperlactatemia identified in 33 cats (46%). When considered as a categorical variable, plasma lactate concentration upon admission was not predictive of survival (*p* = 0.59) (Table 2) nor the length of hospitalization (*p* = 0.58) (Table 3). Similarly, when considered as a continuous variable, plasma lactate concentration upon admission was not predictive of survival (*p* = 0.73) (Table 2) nor the length of hospitalization (*p* = 0.96) (Table 3).

**Table 2:** Prognostic value of admission lactate concentration, recheck lactate concentration, and percent change in lactate concentration in cats presented with respiratory distress to predict outcome (survival vs. non-survival).

	**Outcome**				***p*-value**
	**Survival**		**Non-survival**		
	***N***	**Results**	***N***	**Results**	
Lactate^*^ (mmol/L)	54	2.5 (0.6–12.8)	17	2.7 (1.1–4.8)	0.73
Lactate^°^ (mmol/L)	30 (55.6%)	≤ 2.5	8 (47.1%)	≤ 2.5	0.59
	24 (44.4%)	> 2.5	9 (52.9%)	> 2.5	
Recheck lactate^*^ (mmol/L)	30	1.9 ± 0.9	9	1.2 ± 0.6	0.06
% Change in lactate (%)	29	19.5 ± 47.6	9	50.4 ± 27.3	0.049

* When lactate is considered as a continuous variable.

° When lactate is considered as a categorical variable with lactate ≤ 2.5 being normal and lactate > 2.5 being hyperlactatemia.

Lactate is evaluated as a continuous variable and as a categorical variable with lactate ≤ 2.5 mmol/L being considered as normal and lactate > 2.5 mmol/L being defined as hyperlactatemia.

**Table 3:** Prognostic value of admission lactate concentration, recheck lactate concentration, and percent change in lactate concentration in cats presented with respiratory distress to predict the length of hospitalization.

	***N***	**Results**	**Length of hospitalization (days)**	***p*-value**
Lactate^*^ (mmol/L)	71	2.5 (0.6–12.8)	0.5–12	0.96
Lactate^°^ (mmol/L)	38	≤ 2.5	0.5–5	0.58
	33	> 2.5	0.5–12	
Recheck lactate^*^ (mmol/L)	39	1.7 ± 0.9	0.5–12	0.0463
% Change in lactate (%)	39	26.6 ± 45.4	0.5–12	0.0045

* When lactate is considered as a continuous variable.

° When lactate is considered as a categorical variable with lactate ≤ 2.5 being normal and lactate > 2.5 being hyperlactatemia.

Lactate is evaluated as a continuous variable and as a categorical variable with lactate ≤ 2.5 mmol/L being considered as normal and lactate > 2.5 mmol/L being defined as hyperlactatemia.

When considered as a continuous variable, plasma lactate concentration was not significantly associated with the underlying etiology for respiratory distress (i.e., cardiac vs. respiratory) (*p* = 0.25) (Table 4). Similarly, plasma lactate concentration was not significantly associated with the underlying localization of the respiratory disease (i.e., upper airway, lower airway, parenchymal, pleural, > 1 location) (*p* = 0.58) (Table 4).

**Table 4:** Prognostic value of admission lactate concentration in cats presented with respiratory distress to predict underlying etiology of respiratory distress (cardiac vs. respiratory).

		***N*, %**	**Lactate**	***p*-value**
Underlying etiology	Cardiac origin	36 (51)	2.5 (0.9–4.9)	0.25
	Respiratory origin	35 (49)	2.5 (0.6–12.8)	
Respiratory subcategories	Upper airway	4 (11)	2.1 (1.1–3)	0.58
	Lower airway	9 (26)	2.5 (1–6.1)	
	Parenchymal	7 (20)	3.6 (1.9–5.7)	
	Pleural	7 (20)	2.9 (1.1–5)	
	>1 localization	8 (23)	2.3 (0.6–2.8)	

Respiratory is further subcategorized into upper airway, lower airway, parenchymal, pleural, and more than one respiratory disease localization. Lactate is evaluated as a continuous variable.

### Prognostic utility of recheck lactate and lactate clearance during hospitalization

Recheck lactate (*n* = 39) within 24 h of admission was 1.7 ± 0.9 mmol/L, with an associated % change of 26.6 ± 45.4%. Although recheck lactate was not predictive of survival (*p* = 0.06), lactate clearance was (*p* = 0.049) (Table 2). Both recheck lactate and lactate clearance were predictive of length of hospitalization (*p* = 0.046, *p* = 0.004, respectively) (Table 3). Percent change in lactate was confirmed to be predictive of length of hospitalization by robust regression where an increase in lactate concentration by 1% was associated with an increased length of hospitalization by 0.0079 days (11.4 min) (*p* = 0.035).

## Discussion

In our population of cats presented to the emergency room for respiratory distress, hyperlactatemia was common, with a prevalence of 46%. This is comparable to findings from previous studies, where hyperlactatemia has been reported in 23–67% of cats presenting to the emergency room with various diseases (14, 18, 19).

Unlike the 2015 study by Reineke et al., none of the evaluated vital parameters were found to correlate with hyperlactatemia (17). Such a difference might be explained by the different feline populations evaluated in each study. While we focused on cats with underlying respiratory and cardiac diseases, Reineke et al. included cats with a variety of underlying diseases [renal, gastrointestinal, endocrine, neoplasia, trauma, sepsis/SIRS, cardiovascular, neurologic, hepatic, respiratory, other (anemia, toxicity, anaphylaxis, unknown)] with only nine cats being classified as having respiratory (*n* = 2) or cardiac diseases (*n* = 7) (17). Moreover, Reineke et al. prospectively evaluated perfusion parameters, including mucous membrane color, capillary refill time, peripheral pulse quality, heart rate, rectal temperature, and systolic blood pressure, finding significantly higher admission lactate concentration in cats with hypothermia, white mucous membrane, abnormal peripheral pulse quality, and hypotension (17). Due to the retrospective nature of our study and the subjectivity of parameters, such as mucous membrane color and CRT, we elected to not submit that data for statistical analysis. The lack of correlation between evaluated vital parameters and lactate concentration in our study may also be attributable to the smaller number of cases in the categories Reineke et al. found to be significant. For example, while Reineke et al. had 43 cats with a systolic blood pressure <90 mmHg, only five cats included in our study were hypotensive, which might have introduced type II errors in our analysis. We also considered the respiratory rate and pulse oximetry as we focused on cats presented with respiratory distress. Previous studies have demonstrated that severe hypoxemia (PaO_2_ 25–40 mmHg, SpO_2_ <75%) is necessary before lactate concentration begins to rise (20, 21). Thus, it is not surprising that no correlation was found between lactate concentration and oxyhemoglobin saturation in this population of cats as only 2/14 had an SPO_2_ ≤ 75%. This last finding should however be interpreted cautiously due to limited number of cats with a SpO_2_ reported. Further prospective studies with a consistent recording of pulse oximetry and/or arterial blood gas are encouraged to further evaluate a possible correlation between lactate concentration and oxygenation.

In our population of cats presented in respiratory distress with underlying respiratory or cardiac diseases, admission lactate concentration was not associated with survival or duration of hospitalization. In contrast, lactate clearance was significantly associated with survival. Similarly, Saint-Pierre et al. found lactate clearance to be significantly associated with survival in 444 cats presenting to the emergency room (14). However, our finding should be interpreted cautiously as survivors had a lactate clearance of 19.5 ± 47.6 and non-survivors had a lactate clearance of 50.4 ± 27.3. One would expect survivors to have higher lactate clearance than non-survivors and not the opposite as demonstrated in our study. The significance of this finding is unknown and might be related to a type II error due to the limited number of patients in the non-survivor group. A significant association with length of hospitalization was identified, where a 1% increase in lactate concentration was correlated with an increased length of hospitalization by 11.4 min. While statistically significant, this finding lacks clinical significance. Such findings add more controversy to the literature on the prognostic utility of lactate in the feline population. It should be noted that our results cannot be compared to the majority of previously published studies on the prognostic utility of lactate in cats as a variety of underlying diseases were then considered (14, 17, 18). One study evaluating 55 cats with congestive heart failure also did not find an association between admission hyperlactatemia and survival (22). Of interest, the authors report a median (range) admission lactate of 2.4 (0.4–8.1), which is comparable to the admission lactate concentration obtained in our population of cats with cardiac disease [2.5 (0.9–4.9)] (22).

In the present study, admission lactate was not significantly associated with the underlying etiology for respiratory distress (i.e., cardiac vs. respiratory). Interestingly, while Redavid et al. included a heterogeneous population of cats, patients were subcategorized based on diseased system (i.e., respiratory, cardiovascular, renal …), 5/123 (4.1%) of which had respiratory diseases and 5/123 (4.1%) had cardiovascular diseases (18). Despite a limited number of cats in these sub-categories, the authors also did not find an association between the primary disease, lactate concentrations, and survival. Similarly, Saint-Pierre et al. did not find admission lactate concentration to be significantly associated with underlying disease in 444 cats presenting to the emergency room (14). While the total number of cats with the underlying respiratory and cardiac disease was not reported in this last study, 13/231 (6%) of cats had a respiratory disease and hyperlactatemia and 20/231 (9%) had cardiac disease and hyperlactatemia. This seems to indicate that, admission lactate concentration is unlikely to help predict the likelihood of respiratory vs. cardiac disease in cats presenting in respiratory distress. Previous studies in humans and animals suggest that severe hypoxemia is necessary to produce an increase in anaerobic metabolism (20, 21). Thus, the degree of hypoxemia was likely not severe enough to affect lactate concentration in our population of cats with respiratory disease.

In contrast with our findings, lactate concentration has been demonstrated to have prognostic value in multiple species with respiratory and cardiac diseases. Hyperlactatemia in people with acute heart failure and acute respiratory distress syndrome has been associated with increased mortality (23–25). Similarly, increasing lactate concentration is associated with increased severity of disease in calves with bovine respiratory disease (26). These differing findings might be related to interspecies lactate metabolism differences. Furthermore, interspecies variation between the underlying cause for heart failure (e.g., congestive/hypoxemic vs. hypoperfusion) may explain why lactate concentration may be prognostic in other species. Lactate has been unreliably reported as a prognostic indicator in the feline population, and we could wonder if clinical research should investigate other markers of perfusion in this population.

This study has several limitations inherent to a retrospective study. No scoring system was implemented to assess the severity of the disease, and the median (range) admission lactate concentration was 2.5 mmol/L (0.6–12.8). The survival rate of 76% in our population of cats, as well as the high euthanasia rate inherent to veterinary medicine, might have hindered the prognostic utility of lactate. Due to the retrospective nature of the study, we also faced variability in the completeness of the medical record and missing data, including mucous membrane color, capillary refill time, pulse oximetry, and recheck lactate, which might have introduced type II error in our findings. A minority of cats (*n* = 5) with previously diagnosed heart disease did not have repeat diagnostic imaging at the time of hospitalization. While these cats had an increased LA:Ao and their respiratory distress resolved with furosemide, it is possible the cause of their respiratory distress could have been attributed to another disease process. The effect of struggling could not be evaluated neither. It has been suggested that azotemia might disturb lactate metabolism and/or clearance in hypotensive dogs (27). It is currently unknown if this holds true in the feline population. We did not look at the renal function in our population of cats; however, we could wonder if underlying renal disease as well as the use of furosemide could have affected admission and serial lactate in our group of cats with underlying cardiac diseases. No scoring system to evaluate the work of breathing associated with respiratory distress could be used, while it could lead to type B hyperlactatemia related to increasing respiratory muscle activity. Lastly, while all patients were reliably categorized as having respiratory or cardiac disease, a definitive diagnosis was not obtained in all cases.

Results of the present study suggest that hyperlactatemia is common in cats presenting to the emergency room in respiratory distress but is not predictive of survival or length of hospitalization. Lactate clearance was found to be significantly associated with survival, although non-survivors had higher clearance than survivors. Thus, like dogs, serial lactate concentrations may be more predicative of survival than a single measurement and may provide a more effective way to monitor response to treatment. Although lactate clearance was also found to be predictive of length of hospitalization, it lacks clinical significance. Furthermore, no difference in admission lactate concentration was identified between cats with underlying respiratory vs. cardiac diseases. Future prospective studies enrolling a larger population of cats with underlying cardiac and respiratory disease and documenting various levels of hypoxemia are needed to evaluate the prognostic utility of lactate in cats with respiratory distress.

## Data availability statement

The raw data supporting the conclusions of this article will be made available by the authors, without undue reservation.

## Author contributions

CG was the primary investigator in obtaining patient data. CG wrote the manuscript with the assistance of LG. Both authors contributed to the article and approved the submitted version.

## Conflict of interest

Author LG is a reviewer of the Journal but only participated in the peer review process as an author. The remaining author declares that the research was conducted in the absence of any commercial or financial relationships that could be construed as a potential conflict of interest.

## Publisher's note

All claims expressed in this article are solely those of the authors and do not necessarily represent those of their affiliated organizations, or those of the publisher, the editors and the reviewers. Any product that may be evaluated in this article, or claim that may be made by its manufacturer, is not guaranteed or endorsed by the publisher.
